# Trust in social media and COVID-19 beliefs and behaviours

**DOI:** 10.1371/journal.pone.0275969

**Published:** 2022-10-13

**Authors:** Nicky Nicholls, Eleni Yitbarek

**Affiliations:** Department of Economics, University of Pretoria, Gauteng, South Africa; University of Georgia, UNITED STATES

## Abstract

The study investigates the relationship between trust in social media and beliefs and preventive behaviours in the context of the COVID-19 pandemic. We surveyed 1008 respondents in South Africa to study how trust in social media relative to other information sources predicts perceived risk and adoption of preventive behaviours. Although engagement with and trust in social media do not predict less adoption of preventive behaviours, trusting information from social media more than information from mass media or scientists is associated with less risk perception from COVID-19 and reduces the adoption of preventive behaviours (including vaccines).

## Introduction

With the rise in the use of social media as a source of information, researchers have started to ask how engagement with social media impacts various aspects of decision making. This is particularly relevant to the COVID-19 pandemic. According to [1], the pandemic has been accompanied by an “infodemic”, a term coined to shed light on the threats of misinformation during the management of crises ([2–4].) That is, people are exposed to too much information, much of which is not accurate.

In this study, we investigate the role of reported trust in information from social media on Covid-19 in South Africa. Specifically, we ask whether trusting information from social media more than information from other sources predicts beliefs and behaviours around COVID-19. Our interest in relative trust stems from the fact that people often engage with more than one type of media. Since the content reported by different media outlets might convey very different messaging (particularly in the context of Covid-19), knowing which type of media people trust most would give insight into the source that would be believed in these cases of contradictory messaging ([5]).

South Africa presents an interesting context for this research, as the country has experienced high levels of COVID-19 infections and has implemented varying levels of regulation around lockdowns. However, COVID-19 vaccine hesitancy has threatened the ability of the country to contain the pandemic during the earlier stage. Using nationally representative data, [6] report that a quarter of the adult population in South Africa expressed vaccine hesitancy in February/March 2021. South Africa’s government targeted getting 67% of the population vaccinated by the end of 2021 (our data was collected in late November 2021). Vaccination rates have fallen far short of this. At the time of the survey, only 42% of South Africans aged 18 and older had been vaccinated (sacoronavirus.co.za/latest-vaccine-statistics).

The country has a high prevalence of social media users. According to World Wide Worx, there were 38.2 million (about 64% of the total population) internet users in South Africa in 2021. This number grew yearly by 1.7 million users (or 4.5%) while active social media users increased by 13.6% from 22 million users in January 2020 to 25 million users in January 2021, suggesting that 42% of South Africans are active users of some form of social media.

We investigate two main research questions: (i) Does trust in information from social media relative to information from other media types predict COVID-19 related behaviours (compliance with COVID-19 lockdown regulations, adoption of prophylactic measures and vaccine adoption) and beliefs about COVID-19 risk? (ii) Does engagement with social media relative to other media types predict these behaviours and beliefs? Our main empirical findings are twofold: First, considering trust in social media in isolation, we note that social media engagement is positively related to preventive behaviours. However, this result is different when we focus on people who trust social media more than they trust mass media (usually more reliable sources of information). Individuals trusting social media more than other news outlets report less perceived risk from COVID-19, are less likely to comply with lockdowns and preventive measures and have lower vaccine adoption. A similar pattern emerges when we consider those who report higher trust in social media information than in scientists. This finding suggests that while engaging with social media might not in itself predict less adoption of preventive behaviours, placing higher trust in information from social media than in more reputable information sources is a concern.

## Literature

Research on social media and trust employs a variety of definitions of trust. One strand of this research considers associations between social media and some form of trust. In a literature review conducted by [7], the authors define generalized trust as “trust between people in general and people who do not know each other that well” (p.518), and report that the majority of the studies they considered (8 out of 10) showed a positive relationship between social media and generalized trust. Looking in more detail at the studies included, it is clear that definitions of both trust and of social media in this literature vary considerably. For example, one of the included studies ([8]) uses digital skills including computer use as a measure of social media, noting that digital skills were associated with generalized trust for some groups, but not for others. Their measure of trust is a commonly used one, included in the 2010 PEW survey, as well as other surveys including the General Social Survey: the response options are “most people can be trusted” and “you can’t be too careful”. Another study ([9]) uses the setting up of and interaction with a website allowing landowners to share information to assist in collective bargaining for a natural gas deal as a measure of social media, and anecdotal reports of increased trust within the landowners group after the creation of the website (together with other interactions). A third study ([10]) used self-reported Facebook use as a measure of social media engagement, and a survey measure based on Rosenberg’s Faith in people scale as a measure of trust. They found that more intense use of Facebook was positively associated with social trust. The general idea behind these positive associations is that social media engagement might increase interpersonal trust by allowing new opportunities for participation and discourse.

Related to this literature, in the context of an increased lack of civility in online interactions (e.g. [11] and references therein), a few studies have considered how trust is impacted by positive and negative online interactions. [11] compared trust game interactions following the presentation of civil or uncivil social media interactions (comment threads) or neutral news articles. Trust games measure trust as the decision to share an amount of money with another player, where the other player has the option of returning some of the money that was shared. Money shared is usually increased by the experimenter, such that money can be shared with an investment motive, but trust is required as the receiver is not obliged to return any of the increased pot of money. Although these authors did not find significant differences in trust with uncivil versus neutral presentations, they did note a positive impact on trust when respondents were presented with civil online discourse. [12] consider social media and political trust, measured using a modified trust game where votes (instead of money) were entrusted to decision makers. The authors varied exposure to positive (cooperative) and negative (partisan) messages (tweets) either from an in- or out-group politician, and found that exposure to tweets from an out-group politician had a detrimental impact on willingness to trust the decision maker with votes. Interestingly, they noted that the negative impact on this measure of trust was more pronounced where the respondents engaged with the tweets by liking, retweeting or responding.

Since the onset of the Covid-19 pandemic, another strand of literature has emerged to consider the link between trust in science and a range of beliefs and behavioural and intentions measures. Studies in this area use self-reported measures of trust in science and of behaviours and beliefs. As [13] points out, science and scientific organisations are usually the source of Covid-19 prevention guidelines and messages informing the public about Covid-19 risks. However, there is an increasing divide between science and society, with rising levels of distrust in science ([14]). Considering trust in science and Covid-19, [13, 15] note that higher reported trust in science predicts greater reported compliance with preventive guidelines. Similarly, [16] argue that compliance intentions are predicted by trust in science across 23 countries. However, compliance intentions do not predict infections in their data. [17] further finds that trust in medical and scientific experts is an important predictor of Covid-19 vaccine adoption.

Other studies have considered either engagement with social media or trust in social media as predictors of behaviour associated with preventing the spread of disease. Here again, self-report measures of trust and of beliefs and behaviours are used. [18] found that social media engagement increased perceived risk by promoting fear and anger in individuals in the MERS-CoV epidemic, while [13] found that higher believed risk from Covid-19 was associated with more preventive behaviours. [19] finds support for this link, noting that social media use predicts the adoption of preventive behaviours through increasing perceived risk of Covid-19 infection. However, other studies suggest that social media might negatively impact other important behaviours, particularly vaccine adoption. [20] notes that conspiracy and anti-vax beliefs are associated with greater reliance on social media for health information, and [21] notes that respondents who engaged with traditional media channels (TV, newspapers, etc.) were more likely to accept a vaccine. In contrast, those who engaged with social media were less likely to do this. This marks an interesting change from the findings of [22], where the authors found higher influenza vaccine uptake among social media (Facebook and Twitter) users.

[23] argues that distrust in science is usually underpinned by conspiracy beliefs and ideologies. Related to COVID-19 information, [24] suggests that the challenge is in ensuring that people get accurate information. Perhaps the difference in the link between social media and vaccine adoption in Covid-19 versus influenza vaccine adoption relates to the accuracy of the information shared on social media for these two vaccines. Reports from the [1, 25] suggest that social media was used to manipulate facts and to spread unproven theories about COVID-19. [26] notes that sources that were unverified and that promoted misinformation on COVID-19 were shared more than those linked to verifiable health sources. This is confirmed by [27] who found that over 25% of the most viewed YouTube videos about COVID-19 included misleading information. Similar findings on sharing of information from unreliable sources are reported in [4].

In the current study, we contribute to the debate around trust in and engagement with social media and how this relates to the adoption of Non-Pharmaceutical Interventions (NPIs), lockdown compliance and vaccination. Social media has become a widespread source of information around COVID-19. Further, the information and recommendations received from social media might differ qualitatively from those in more traditional media outlets. We, therefore, consider whether different beliefs and behaviours are observed among respondents who engage with social media more than other media channels. We hypothesise that people who trust social media information more than mainstream media practice less preventive behaviour, adopt more risky behaviour, and will be more COVID-19 vaccine-hesitant. Our work is related to [5], which examines whether American’s media preferences (CNN and Fox News) are associated with Covid mitigating health behaviour. Since precise definitions of trust vary in the literature, we follow existing approaches to asking about trust, including [28] for trust in different media types and [15] for trust in science and for general trust in social media.

## Materials and methods

### Respondents

To ensure a reasonable sample size of respondents from different demographic groups, we used a online sample of 1008 respondents in South Africa, recruited through an online survey panel provider, TGM Research. (TGM recuits panelists using email, mobile apps, referrals, marketing campaigns and social media. The company uses a range of data quality assessment tools, including advanced digital finger printing and multifactor screening.) Participants had to be living in South Africa at the time of the survey and to be aged 18 years or older. The online data collection took place from 24 to 26 November, 2021.

The start of the online survey was an informed consent page, where respondents were provided with some information about the study. This information ended with an option to continue with the survey, preceded by the text, “By continuing with the survey you confirm that: You have read and understand the information provided above; You give your consent to participate in the study on a voluntary basis.” Participants could leave the survey at this point if they did not wish to continue. Full details of the questionnaire, including the informed consent, are included in the S1 Questionnaire. The study was approved by the University of Pretoria Economic and Management Sciences ethics committee: EMS241/41.

### Questionnaire

Recall that each of our research questions had COVID-19 related beliefs and preventive behaviours as dependent variables. To investigate COVID-19 related behaviours we include three questions, giving us three different measures for this variable: (i) reported compliance with government regulations around lockdown (measured on a scale from 0 “not at all” to 10 “I follow all regulations all of the time”); (ii) reported compliance with recommended behaviours aimed at preventing the spread of Covid-19 (mask wearing, hand washing, social distancing, etc.) measured on a scale from 0 “not at all” to 10 “I follow all these recommendations all of the time”; and (iii) vaccine adoption (we ask if respondents have been vaccinated, and whether they have been fully vaccinated or received only one dose of Pfizer, the only 2-dose vaccine available in South Africa at the time of the survey). At the time of data collection, all South Africans aged 18 and over (our sample was limited to respondents in this age group) had access to either the 2-dose Pfizer (BioNTech) vaccine, or the single dose Johnson & Johnson (Janssen) vaccine.

Our second dependent variable is beliefs about COVID-19 risk. To measure these beliefs, we use a 6-item survey from [13]. Responses to each item are on a 7-point Likert scale, ranging from “disagree strongly” to “agree strongly”. Responses are coded from 0 to 6, such that respondents have an index of COVID-19 risk perception ranging from 0 (lowest perceived risk level) to 36 (highest perceived risk level) across the 6 questions.

Our explanatory variables are metrics of media engagement and trust in different media types. Our primary variables of interest relate to trust in and engagement with social media relative to other media types. For our main analysis, we use Twitter, Facebook and WhatsApp as a proxy for social media. As a robustness check, we also use a broader definition, where we simply ask respondents about how much they think social media can be trusted, without stipulating a specific social media definition. To measure trust and engagement, we adapt questions from [28]. We ask respondents “Which of the following news sources do you use to get your news? “with options” Twitter, Facebook or WhatsApp”, “Local (South African) TV or Radio News”, “Local (South African) print or online newspapers (e.g. News 24, Mail & Guardian, IOL, Business Day, etc.)” and “International TV or online news”. Respondents can choose all outlets that they use. We then ask a series of questions for each of the four outlet types, where the order of the outlet types is randomised for each question. Our main questions are “How much trust and confidence do you have in each of the following sources when it comes to reporting about COVID-19 fully, accurately and fairly?” (possible answers are “none at all”, “not very much”, “a fair amount” and “a great deal”) and “How frequently do you get news and information from each of the below sources about COVID-19?” (answers are “never”, “rarely/hardly ever”, “sometimes” and “often”). For these questions, respondents could also say that they were not familiar with the outlet type.

As an alternative explanatory variable, we measure trust in science and trust in social media. We use questions from [15] for these measures. Respondents were asked how much they think each source (scientists and social media) can be trusted, where answer options are “not at all”, “a small amount/occasionally”, “mostly”, and “completely”. The order of the question is randomised to reduce any possible bias from the question order.

Finally, we include a series of demographic questions which we use as control variables in our estimates: age, gender, income, general health, education level and presence of children or elderly people in the home.

Given concerns about attention in online surveys, we include two methods to confirm that respondents are answering attentively. Our screener included a question where respondents had to select an age range (those selecting under 18 were not included in the survey, since our ethics approval was for respondents aged 18 and over). In the demographic section near the end of the questionnaire, respondents were asked to report their age in years. Any respondents for whom the reported age did not match the age range selected in the screener were excluded from the final data (n = 13). Following [29], we also included an attention check question as one of our initial screening questions: “Many people enjoy watching or playing different sports, and most have a favourite. We would like to know about your favourite sport, but we also want to check that you read questions carefully. To show that you have read this question properly, please ignore the following question and simply choose tennis. What is your favourite sport?” A list of sports followed, including tennis. Respondents who did not pass this attention check were terminated from the survey and thanked for their time.

### Estimation strategy

To answer our two research questions, we estimate the following model, first for our behaviour measures, and then for our beliefs measure:

$$\begin{array}{c} Behaviour_{i}=\alpha_{0}+\beta_{1}SMEngagement_{i}+\beta_{2}SMTrust_{i}+\gamma X+\epsilon \end{array}$$ (1)

We estimate 3 versions of this model for each of the 3 measures of *Behaviour* for individual *i*: reported compliance with lockdown regulations; reported adoption of recommended COVID-19 prophylactic measures, and vaccination status (vaccinated or not).

We repeat estimates of Eq 1 with *Beliefs* as the dependent variable to see how the same measures predict beliefs about COVID-19 risk. Remember that to measure beliefs about COVID-19 risk, we included a 6-item questionnaire in which respondents report 7-point Likert scale responses, ranging from “disagree strongly” to “agree strongly” for each question. Thus, belief is an index of COVID-19 risk perception ranging from 0 (lowest perceived risk level) to 36 (highest perceived risk level) across the 6 questions.

Our *SMEngagement* measure considers the frequency of engagement with social media for news about COVID-19 relative to the frequency of engagement with other media outlets. For our main regressions, this is coded as 1 for respondents who report more frequent engagement with social media than with all of the other media types considered (local TV/radio news; local print/online news and international news); and 0 otherwise. For example, a respondent whose reported frequency of engagement with social media was higher than their reported frequency of engagement with local TV/radio news, but lower than their reported frequency of engagement with local print/online news, would be coded as 0 (social media is not the media type they engage with most frequently). Conversely, a respondent whose reported frequency of engagement with social media was higher than their reported frequency of engagement with local TV/radio news AND higher than their reported frequency of engagement with local print/online news AND higher than their reported frequency of engagement with international news would be coded as 1 (social media is the media type they engage with most frequently). We also include robustness checks with different formulations of our independent variables, detailed in the robustness section.

Similarly, our *SMTrust* measure compares trust in social media news with trust in news from other outlets. Again, this is coded as 1 where reported trust in social media about COVID-19 news is higher than reported trust in other news outlets; and 0 otherwise. We also consider trust in social media relative to trust in scientists as an alternative measure of trust. This is coded as 1 where reported trust in social media is higher than reported trust in scientists, and 0 otherwise. **X** is a vector of control variables: gender, age, race, self-reported health rated as “very good” or “excellent”; whether the respondent lives with children under 18; whether the respondent lives with an elderly person; whether the respondent holds a degree; and whether the respondents’ household income is below the 50th percentile for South Africa (ZAR 3,850 per month). The 50th percentile for household income was calculated based on data from the 2017 South African Labour Force Survey, adjusted for inflation.

### Data

Table 1 summarises our sample data. 4 respondents reported a different gender (neither male nor female). One respondent preferred not to disclose gender and 7 respondents preferred not to disclose race. 50 respondents chose not to disclose their household income: the response option not to disclose income included “don’t know / prefer not to say”.

**Table 1:** Demographic summary and main outcome measures.

	Total Sample	Trust SM Most	Not SM Most
**Categorical variables (%)**			
Gender: Male/Female	49.3/50.2	44.9/51.0	50.5/49.2
Race: Black	67.5	71.4	67.3
Race: White	17.4	16.3	17.4
Race: Other	14.5	12.2	15.3
Low HH Income: less than ZAR 3,850 monthly*	11.6	20.4	11.2
Children under 18 in home (yes = 1)	59.2	63.3	59.0
Live with Elderly person (yes = 1)	32.2	36.7	32.0
Degree (1 = Bachelor’s degree or higher)	59.8	59.2	59.9
Healthy (very good or excellent health = 1)	62.9	57.1	63.2
Vaccinated against Covid-19 (yes = 1)	56.6	20.4	58.4
Trust Social Media most	4.9	1	0
Highest Frequency SM	6.5	26.5	5.4
Trust SM over scientists	8.2	26.5	7.3
Trust SM as much as/more than others	36.9	1	33.7
Frequency SM as much as/more than others	58.7	89.8	57.1
**Continuous variables: Mean (s.d.)**			
Age (in years)	34.75 (10.92)	34.31 (11.00)	34.78 (10.92)
Measures of beliefs and behaviour:			
Perceived Covid-19 risk (scale 0 to 36)	20.74 (6.46)	18.14 (8.65)	20.88 (6.31)
Lockdown compliance (scale 0 to 10)	8.16 (1.95)	6.81 (2.54)	8.23 (1.90)
Prophylactic adoption (scale 0 to 10)	8.07 (1.96)	6.92 (2.71)	8.12 (1.90)
General SM Trust	1.049 (0.66)	1.27 (0.64)	1.038 (0.66)
SM Trust for Covid-19	1.54 (0.92)	2.29 (0.61)	1.50 (0.91)
SM Frequency for Covid-19	2.33 (0.86)	2.73 (0.49)	2.31 (0.87)
n	1008	49	959

*Income data was gathered in broad categories, full questionnaire is included in the S1 Questionnaire.

Relative to the South African population, where 79% of people are black (Census, 2011), our sample has fewer black respondents (68%) and fewer low income households (only 12% of our sample is below the 50th income percentile). Our sample is also more likely to be educated: 60% of our sample reported having a degree, versus 6% of the population holding university degrees, and another 6% holding diplomas (https://www.dhet.gov.za). The vaccination rate of 57% in our sample was also higher than that in South Africa at the time of the survey: at that time, 42% of South Africans aged 18 and over had been vaccinated (sacoronavirus.co.za/latest-vaccine-statistics). The demographic skews in the sample are related to the online sampling methodology used.

**Fig 1:**
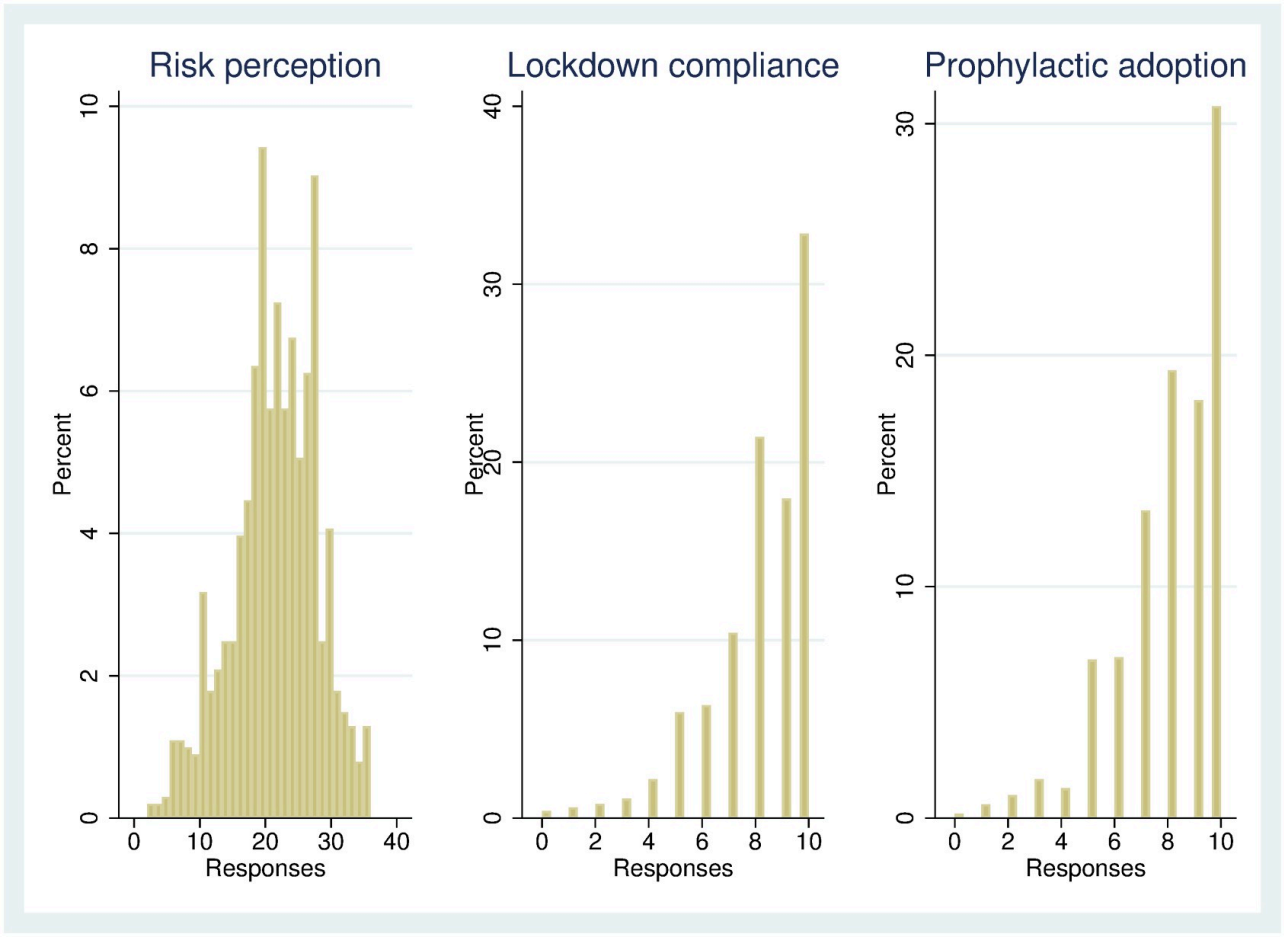
Distribution of responses to beliefs and behaviour measures.

The distributions for the three measures of beliefs and behaviour are shown in Fig 1. We note a significant probability mass (over 30%) at 10 (responses ranged from 0 to 10) for the lockdown compliance and prophylactic measures. For ease of interpretation, we use OLS estimates with robust standard errors for our main estimates. As robustness checks, we also use Tobit estimates for the lockdown compliance and prophylactic measures.

## Results

Table 2 presents the main results, with four columns reporting estimates for each of the four outcome measures described in the Estimation section. Our first research question referred to trust in social media relative to other media types. Recall that we used a dummy variable indicating higher social media trust versus local TV/radio; local print/online and international media. Respondents who trust social media more than the other media outlets report significantly lower believed risk from COVID-19; and are also significantly less likely to adopt behaviours aimed at preventing the spread of the virus (compliance with lockdown regulations and adoption of prophylactic measures). These respondents are also significantly less likely to report having been vaccinated against COVID-19.

**Table 2:** OLS estimates: Social media, beliefs and behaviour.

	(1)	(2)	(3)	(4)
	Beliefs	Lockdown Compliance	Prophylactic Adoption	Vaccinated (logit)
**Trust Social Media most**	-2.599**	-1.294***	-1.104***	-1.649***
	(1.165)	(0.370)	(0.390)	(0.374)
**Highest Frequency SM**	-1.965**	-0.404	-0.397	-0.199
	(0.791)	(0.315)	(0.336)	(0.275)
Female	1.280***	0.651***	0.738***	0.0481
	(0.396)	(0.123)	(0.124)	(0.134)
White	-2.237***	-0.464*	-0.278	-0.130
	(0.744)	(0.240)	(0.213)	(0.237)
Black	-1.255**	-0.152	-0.335**	0.0187
	(0.596)	(0.164)	(0.167)	(0.194)
Age (in years)	0.0644***	0.0309***	0.0230***	0.0149**
	(0.0214)	(0.00642)	(0.00614)	(0.00717)
Healthy	-2.968***	0.256**	0.345***	-0.00708
	(0.394)	(0.123)	(0.123)	(0.138)
Live with kids Under 18	0.904**	0.140	-0.00209	-0.0254
	(0.412)	(0.132)	(0.127)	(0.142)
Live With Elderly	0.0874	-0.191	-0.0251	0.102
	(0.416)	(0.132)	(0.125)	(0.143)
Low Income	1.008*	0.0919	0.142	-0.592***
	(0.603)	(0.206)	(0.206)	(0.216)
Degree (Bachelor’s degree or higher)	0.698	0.129	0.131	0.455***
	(0.425)	(0.134)	(0.134)	(0.142)
Constant	20.14***	6.771***	6.952***	-0.394
	(1.078)	(0.292)	(0.299)	(0.350)
N	1008	1008	1008	1008
R-sq/chi-sq	0.098	0.083	0.069	48.28***

Standard errors in parentheses

* p<0.10;

** p<0.05;

*** p<0.010

Notes: These results are robust to the inclusion of geographical location (province) and political party supported as additional controls. These results are available from the authors on request.

Our second research question considered the frequency of engagement with social media relative to other media types. We note a significant negative relationship between believed risk from COVID-19 and engaging more with social media than other news. Engaging most with social media predicts neither compliance with lockdown regulations nor the adoption of prophylactic measures. The likelihood of being vaccinated is also not predicted by our measure of engagement with social media.

As discussed earlier in the Materials and Methods section we use OLS with robust standard errors for most of our main estimates for ease of interpretation. Since vaccination status is a binary variable, we use a Logit model for this main estimation. The OLS version of this model is included in the appendix for ease of interpretation. To check the robustness of our OLS results, in the appendix, we report Tobit estimates for the lockdown compliance and prophylactic measures since both have significant probability mass (over 30%) at 10 (responses ranged from 0 to 10). The qualitative results in the appendix remain unchanged; although engagement with and trust in social media are not associated with less adoption of preventive behaviours, trusting information from social media more than information from mass media or from scientists is associated with less risk perception and reduced adoption of preventive behaviours (see S2 Table).

### Robustness to alternative measures

In Table 3, we consider alternative measures of engagement and trust in social media to check whether our results are robust to different specifications for these constructs. For ease of comparison, we simply report the coefficients for the variables of interest from the regressions. All regressions include the reported variables as well as the control variables, detailed in Table 2. We start by reporting the main measures from Table 2 as a benchmark. We then introduce a measure comparing respondents’ reported trust in social media to their reported trust in scientists. This variable takes a value of 1 where the respondent reports higher trust in social media than in scientists, and 0 otherwise. We find similar results to our main trust variable for beliefs about COVID-19 risk; and directionally similar, but smaller in magnitude, results where our 3 measures of behaviour are considered (lockdown compliance, prophylactic adoption and vaccination).

**Table 3:** Comparing impacts of different measures of trust and frequency.

	(1)	(2)	(3)	(4)
	Beliefs	Lockdown Compliance	Prophylactic Compliance	Vaccinated (logit)
**Main measures for Covid-19**				
Trust Social Media most	-2.599**	-1.294***	-1.104***	-1.649***
	(1.165)	(0.370)	(0.390)	(0.374)
Highest Frequency SM	-1.965**	-0.404	-0.397	-0.199
	(0.791)	(0.315)	(0.336)	(0.275)
**Trust across sources**				
Trust SM over Scientists	-3.434***	-0.652**	-0.678**	-0.858***
	(0.747)	(0.261)	(0.265)	(0.247)
**Including equal or greater trust and freqeuncy for SM versus other media**				
Trust SM equal or greater	-1.815***	-0.350**	-0.0792	-0.417***
	(0.444)	(0.139)	(0.136)	(0.144)
Frequency SM equal or greater	0.122	0.0655	-0.0534	-0.0838
	(0.414)	(0.127)	(0.124)	(0.142)
**Trust and Frequency for SM alone**				
Trust SM for Covid-19	0.047	0.176**	0.349***	0.291***
	(0.248)	(0.0751)	(0.0788)	(0.0804)
Frequency SM for Covid-19	0.431*	0.0827	-0.0449	-0.144*
	(0.257)	(0.0863)	(0.0787)	(0.0851)
**General trust in Social Media**				
Trust in Social Media	0.107	0.229**	0.348***	0.206**
	(0.319)	(0.0923)	(0.0917)	(0.0996)

Standard errors in parentheses

* p<0.10;

** p<0.05;

*** p<0.010

Notes: “Trust SM over Scientists” considers whether reported trust in social media is greater than reported trust in scientists; “Trust SM equal or greater” and “Frequency SM equal or greater” define whether trust in social media or frequency of social media use are at least as high as trust in other outlets or frequency of use of other outlets. Like the main measures, these are all dummy variables taking the value of 0 or 1. The final 3 variables are simply measures for social media (rather than comparisons between the measures for social media versus the measures for other media/scientists). These are therefore categorical variables, converted to a scale from 0 to 3: “Trust SM for Covid-19” considers reported trust in social media for Covid-19 information; while “Frequency SM for Covid-19” considers reported frequency of engagement with social media for Covid-19; “Trust in Social Media” reports overall trust in social media.

Since the number of respondents reporting higher trust in social media (n = 49) or frequency of engagement with social media (n = 83) than all other news outlets is relatively small, we also consider a weaker measure for relative trust and engagement. Here we code respondents as highly trusting of (n = 372) or engaging with (n = 592) social media if their reported trust or frequency of social media engagement is at least as high as that for the highest other news outlet. Overall, we find directionally similar results using this measure, although magnitudes of these coefficients are, unsurprisingly, smaller. This frequency measure is no longer a significant predictor of beliefs around Covid-19 risk.

Finally, we are interested in whether the degree of trust in social media or the frequency of use of social media predict these outcomes (recall that our previous measures used social media trust or frequency relative to other media types). These are our final 3 measures reported in Table 3. Interestingly, our 2 trust measures (degree of trust in social media for accurate reporting on COVID-19 and degree of general trust in social media) both show positive relationships with our 3 measures of behaviour. This finding suggests that higher trust in social media does not necessarily predict less caution around Covid-19. Rather, it seems that trusting social media more than other news outlets or more than scientists is associated with fewer mitigating behaviours for COVID-19.

As in Table 2, we use OLS estimation with robust standard errors for the main estimates with the exception of the logit estimate for vaccine adoption, and replicate the same table in the S3 Table using Tobit regressions for the lockdown compliance and prophylactic adoption dependent variables; and an OLS regression for ease of interpretation for the vaccinated dependent variable.

## Discussion

To date, South Africans’ adoption of vaccines has not matched the availability of vaccine doses in the country. The number of COVID-19 cases in the country has been high, and vaccination and adoption of preventive behaviours remain the best ways to reduce the spread of the virus. With high social media outreach in the country, disseminating information (and hence, potentially, misinformation) has become easier than ever before. (South Africa has the fourth-highest number of internet users in Africa: https://www.statista.com/statistics/505883/number-of-internet-users-in-african-countries/). Compared with mass media, social media offers an unprecedented opportunity to spread false narratives about the virus and the vaccine. To date, limited research investigating the link between social media engagement and trust and adoption of COVID-19 preventive measures has shown mixed results. The South African context makes this country a good setting to further study this relationship.

This study considered how COVID-19 preventive behaviour is related to trust in social media relative to trust in other media types and relative to trust in scientists. Our findings make an interesting contribution to the literature. While trust in social media, either generally or as a source of information about COVID-19, shows a small but significant positive correlation with preventive health behaviours (compliance with lockdown regulations, adopting prophylactic behaviours and getting vaccinated), this result is very different when trust in social media exceeds trust of other, more reputable media sources. Respondents who trust social media more than other news outlets for COVID-19 information perceive less risk from COVID-19 and are also significantly less likely to comply with lockdown regulations or adopt behaviours aimed at reducing the likelihood of infection. This is also true for those who report trusting social media over scientists.

The findings suggest that engagement with social media is not associated with more risky behaviours. Indeed, our results show that those who engaged more with social media had higher risk perceptions and did not show less compliance to preventive behaviours. Instead, it seems that trusting social media more than scientists or traditional media outlets is associated with less adoption of preventive behaviours. Since respondents who trusted social media over other sources of information also report lower perceived risk from COVID-19, lower risk perceptions present a likely mechanism for explaining the reduced adoption of preventive behaviours.

Our study has important limitations to note. The use of online surveys, like ours, tends to have relatively low representation of certain population groups, particularly older, lower income and less educated respondents. Further, inattention among respondents can limit the quality of data. We attempted to mitigate inattention concerns by using an attention check question, and excluding those who did not correctly answer this question. Data from surveys such as the one used for this study are cross-sectional, and causal inferences can therefore not be drawn from correlations between variables. We also note that our measures of lockdown compliance and prophylactic adoption are single item measures, which carry a risk of low reliability. Our multi-item beliefs measure shows fairly low internal consistency (*alpha* = 0.54), likely related to the small number of items included in this measure. Further research could consider robustness of our findings to alternative measures for these beliefs and behaviours. Finally, we ask only broadly about trust and engagement with social media and other media types. We do not ask about the specific media content with which people engage on the different media platforms. This, too, could be investigated in more detail in future research.

## Supporting information

S1 Table Details of responses to categorical predictor variables. (PDF)

S2 Table Regressions using Tobit and logit models for behaviour. (PDF)

S3 Table Comparing impacts of different measures using Tobit and OLS models. (PDF)

S1 Questionnaire Informed consent and questionnaire. (PDF)
