# Supplementary material for: Trust in social media and COVID-19 beliefs and behaviours
Source: PLoS One. 2022 Oct 13;17(10):e0275969. doi: 10.1371/journal.pone.0275969 (PMC9560499; doi:10.1371/journal.pone.0275969)
Supplement: S1 Table — (PDF) [file pone.0275969.s001.pdf]

S1 Table. Details of responses to categorical predictor variables

	0*	1**	2***	3****
**Trust SM for Covid-19**	13.31	35.85	34.76	16.09
**Frequency SM for Covid-19**	5.09	10.58	30.64	53.69
**General Trust SM**	17.56	62.10	18.25	2.08

*respectively, "none at all"; "never"; "not at all"
**respectively, "not very much"; "rarely/hardly ever"; "a small amount/occasionally"
***respectively, "a fair amount"; "sometimes"; "mostly"
****respectively, "a great deal"; "often"; "completely"
