# Supplementary material for: Trust in social media and COVID-19 beliefs and behaviours
Source: PLoS One. 2022 Oct 13;17(10):e0275969. doi: 10.1371/journal.pone.0275969 (PMC9560499; doi:10.1371/journal.pone.0275969)
Supplement: S2 Table — (PDF) [file pone.0275969.s002.pdf]

S2 Table. Regressions using Tobit and Logit models for behaviour

	(1) Beliefs (OLS)	(2) Lockdown Compliance (Tobit)	(3) Prophylactic Adoption (Tobit)	(4) Vaccinated (OLS)
**Trust Social Media most**	-2.599**	-1.615***	-1.414***	-0.357***
	(1.165)	(0.448)	(0.467)	(0.0632)
**Highest Frequency SM**	-1.965**	-0.568	-0.398	-0.0442
	(0.791)	(0.402)	(0.435)	(0.0630)
Female	1.280***	0.924***	1.050***	0.0119
	(0.396)	(0.178)	(0.174)	(0.0312)
White	-2.237***	-0.689**	-0.492	-0.0309
	(0.744)	(0.341)	(0.309)	(0.0555)
Black	-1.255**	-0.155	-0.471*	0.00350
	(0.596)	(0.250)	(0.253)	(0.0451)
Age	0.0644***	0.0514***	0.0368***	0.00346**
	(0.0214)	(0.00979)	(0.00901)	(0.00164)
Healthy	-2.968***	0.397**	0.571***	-0.00262
	(0.394)	(0.174)	(0.169)	(0.0322)
Kids Under 18	0.904**	0.229	0.0237	-0.00460
	(0.412)	(0.188)	(0.180)	(0.0330)
Live With Elderly	0.0874	-0.197	-0.0531	0.0239
	(0.416)	(0.186)	(0.175)	(0.0333)
Low Income	1.008*	0.217	0.348	-0.140***
	(0.603)	(0.289)	(0.295)	(0.0503)
Degree	0.698	0.171	0.118	0.107***
	(0.425)	(0.188)	(0.184)	(0.0336)
Constant	20.14***	6.385***	6.810***	0.411***
	(1.078)	(0.430)	(0.428)	(0.0816)
N	1008	1008	1008	1008
R-sq/F	0.098	9.93***	7.89***	0.048

Standard errors in parentheses

* $p<0.10$; ** $p<0.05$; *** $p<0.010$
