# Supplementary material for: Trust in social media and COVID-19 beliefs and behaviours
Source: PLoS One. 2022 Oct 13;17(10):e0275969. doi: 10.1371/journal.pone.0275969 (PMC9560499; doi:10.1371/journal.pone.0275969)
Supplement: S3 Table — (PDF) [file pone.0275969.s003.pdf]

S3 Table. Comparing impacts of different measures using Tobit and OLS models

	(1) Beliefs (OLS)	(2) Lockdown Compliance (Tobit)	(3) Prophylactic Compliance (Tobit)	(4) Vaccinated (OLS)
**Main measures for Covid-19**				
Trust Social Media most	-2.599**	-1.615***	-1.414***	-0.357***
	(1.165)	(0.448)	(0.467)	(0.0632)
Highest Frequency SM	-1.965**	-0.568	-0.398	-0.0442
	(0.791)	(0.402)	(0.435)	(0.0630)
**Trust across sources**				
Trust SM over Scientists	-3.434***	-0.841**	-0.833**	-0.203***
	(0.747)	(0.348)	(0.342)	(0.0564)
**Including equal or greater trust and freqeuncy for SM versus other media**				
Trust SM equal or greater	-1.815***	-0.421**	-0.0170	-0.0997***
	(0.444)	(0.199)	(0.189)	(0.0347)
Frequency SM equal or greater	0.122	0.0984	-0.0852	-0.0194
	(0.414)	(0.184)	(0.174)	(0.0335)
**Trust and Frequency for SM alone**				
Trust SM for Covid-19	0.047	0.247**	0.505***	0.0687***
	(0.248)	(0.111)	(0.110)	(0.0186)
Frequency SM for Covid-19	0.431*	0.0927	-0.0698	-0.0339*
	(0.257)	(0.120)	(0.105)	(0.0200)
**General trust in Social Media**				
Trust in Social Media	0.107	0.298**	0.501***	0.0485**
	(0.319)	(0.139)	(0.134)	(0.0234)

Standard errors in parentheses
* p<0.10; ** p<0.05; *** p<0.010
