# Supplementary material for: Trust in social media and COVID-19 beliefs and behaviours
Source: PLoS One. 2022 Oct 13;17(10):e0275969. doi: 10.1371/journal.pone.0275969 (PMC9560499; doi:10.1371/journal.pone.0275969)
Supplement: S1 Questionnaire — (PDF) [file pone.0275969.s004.pdf]

Trust in social media and COVID-19 beliefs and behaviours

Informed consent and questionnaire

Informed consent

Dear Participant, Thank you for your willingness to participate in this survey on media engagement and perceptions and behaviours associated with Covid-19. This project is being conducted by researchers at the University of Pretoria.

All of your responses will be completely anonymous and will be used for research purposes only. There is no right or wrong answer to any of the questions that you are asked to answer. We simply ask you to respond honestly, by giving the answer that best shows your preference or opinion.

We anticipate that the survey will take about 10 minutes to complete. Please note that your participation is voluntary and there will be no penalty or loss of benefit if you decide not to participate. You are free to stop participating at any point without having to explain your decision. If you would like to ask questions before participating, you are welcome to do so by emailing experimentaleconomicsresearch@gmail.com. Finally, note that as a participant, you have the right of access to your data.

By continuing with the survey you confirm that:

- You have read and understand the information provided above.
- You give your consent to participate in the study on a voluntary basis.

Start of Block: screener

How old are you?

- ○ 12-17 years (*terminate*)
- ○ 18-24 years
- ○ 25-29 years
- ○ 30-34 years
- ○ 35-39 years
- ○ 40-44 years
- ○ 45-49 years
- ○ 50-54 years
- ○ 55-59 years
- ○ 60 years or older

Where do you currently live?

- ○ Botswana (*terminate*)
- ○ South Africa
- ○ Zimbabwe (*terminate*)
- ○ Other African country (*terminate*)
- ○ I don't currently live in Africa (*terminate*)

Many people enjoy watching or playing different sports, and most have a favourite. We would like to know about your favourite sport, but we also want to check that you read questions

carefully. To show that you have read this question properly, please ignore the following question and simply choose tennis. What is your favourite sport?

- ○ Soccer (*terminate*)
- ○ Rugby (*terminate*)
- ○ Tennis
- ○ Hockey *(terminate*)
- ○ Swimming (*terminate*)
- ○ Other (*terminate*)

End of Block: screener

Which of the following news sources do you use to get your news? (please select all sources that you use)

- ▢ Twitter, Facebook or WhatsApp
- ▢ Local (South African) TV or Radio News
- ▢ Local (South African) print or online newspapers (e.g. News 24, Mail & Guardian, IOL, Business Day, etc.)
- ▢ International TV or online news (please specify preferred news outlet) ____

How much trust and confidence do you have in each of the following sources when it comes to reporting about politics and current events fully, accurately and fairly?

	None at all	Not very much	A fair amount	A great deal	I'm not familiar with these outlets
Twitter, Facebook and WhatsApp	○	○	○	○	○
Local (SA) TV or Radio News	○	○	○	○	○
Local (SA) print or online newspapers (e.g. News 24; Mail & Guardian; IOL; Business Day, etc.)	○	○	○	○	○
International TV or online news	○	○	○	○	○

How frequently do you get news and information from each of the below sources about politics and current events?

	Never	Rarely / hardly ever	Sometimes	Often	I'm not familiar with these outlets
Twitter, Facebook and WhatsApp	○	○	○	○	○
Local (SA) TV or Radio News	○	○	○	○	○
Local (SA) print or online newspapers (e.g. News 24; Mail & Guardian; IOL; Business Day, etc.)	○	○	○	○	○
International TV or online news	○	○	○	○	○

How much trust and confidence do you have in each of the following sources when it comes to reporting about COVID-19 fully, accurately and fairly?

	None at all	Not very much	A fair amount	A great deal	I'm not familiar with these outlets
Twitter, Facebook and WhatsApp	○	○	○	○	○
Local (SA) TV or Radio News	○	○	○	○	○
Local (SA) print or online newspapers (e.g. News 24; Mail & Guardian; IOL; Business Day, etc.)	○	○	○	○	○
International TV or online news	○	○	○	○	○

How frequently are you getting news and information about COVID-19 from each of the below sources?

	Never	Rarely / hardly ever	Sometimes	Often	I'm not familiar with these outlets
Twitter, Facebook and WhatsApp	○	○	○	○	○
Local (SA) TV or Radio News	○	○	○	○	○
Local (SA) print or online newspapers (e.g. News 24; Mail & Guardian; IOL; Business Day, etc.)	○	○	○	○	○
International TV or online news	○	○	○	○	○

How closely would you say that you follow government regulations around lockdown? Please answer using a scale of 0 to 10, where 0 means "not at all" and 10 means "I follow all regulations all of the time". Please simply drag the slider to indicate your answer.

	Following lockdown regulations
	0 1 2 3 4 5 6 7 8 9 10
Please drag the dot to your chosen answer	

Consider the following recommendations: keeping a 1.5m distance from others, coughing or sneezing into an elbow, washing your hands regularly, wearing a face mask outside your home, avoiding touching your face, avoiding seeing friends/relatives socially, self-isolating after being in contact with someone who has tested positive for COVID-19.

How closely would you say that you follow the above recommendations? Please answer using a

scale of 0 to 10, where 0 means "not at all" and 10 means "I follow all these recommendations all of the time". Please simply drag the slider to indicate your answer.

	Following the listed recommendations<br>0 1 2 3 4 5 6 7 8 9 10
Please drag the dot to your chosen answer	

Have you already received a vaccine for COVID-19?

- ◯ Yes, fully vaccinated (e.g. one dose of J&J or 2 doses of Pfizer)
- ◯ Yes, but only one dose of the Pfizer vaccine
- ◯ No

What is your main reason for not having a COVID-19 vaccine?

- ◯ Religious reasons
- ◯ I don't trust the science
- ◯ I don't trust the government
- ◯ No knowledge of long term side effects
- ◯ I know someone who had bad side effects
- ◯ I've read about people who had bad side effects
- ◯ I don't have access to the vaccine
- ◯ I'm waiting for different vaccines to be available in South Africa (if selected, please say which vaccine you would prefer)
- ◯ I don't believe COVID-19 exists or is serious enough for a vaccine
- ◯ Other reason (please specify)

How much do you think you can trust scientists?

- ◯ Not at all
- ◯ A small amount/occasionally
- ◯ Mostly
- ◯ Completely

How much do you think you can trust the South African government?

- ◯ Not at all
- ◯ A small amount/occasionally
- ◯ Mostly
- ◯ Completely

How much do you think you can trust social media?

- ◯ Not at all
- ◯ A small amount/occasionally
- ◯ Mostly
- ◯ Completely

Please indicate your (dis)agreement with each of the following COVID-19-related items

	Strongly disagree	Disagree	Somewhat disagree	Neither agree nor disagree	Somewhat agree	Agree	Strongly agree
I feel vulnerable to COVID-19 infection	○	○	○	○	○	○	○
I believe there is a chance that my family members will get infected with COVID-19	○	○	○	○	○	○	○
It is extremely unlikely that I will get infected with COVID-19	○	○	○	○	○	○	○
Picturing myself getting COVID-19 is something I find very hard to do	○	○	○	○	○	○	○
I believe that COVID-19 poses a serious threat	○	○	○	○	○	○	○
I worry about getting infected with COVID-19	○	○	○	○	○	○	○

How old are you? Please simply type your current age (years) in the block.

What is your gender?

- ◯ Male
- ◯ Female
- ◯ Other / non-binary
- ◯ Prefer not to say

How many children under the age of 18 do you have?

- ◯ none
- ◯ 1
- ◯ 2
- ◯ 3
- ◯ 4
- ◯ 5 or more

What is the highest level of education that you have completed?

- ◯ Some primary school (no high school)
- ◯ Some high school, but not finished matric
- ◯ Matric
- ◯ Undergraduate degree/diploma
- ◯ Postgraduate degree

What is your total monthly household income after tax? Please include wages, salary, bonuses, tips, any income from your own businesses, etc.

- ◯ Less than R2,000 per month
- ◯ Between R2,000 and R3,849 per month
- ◯ Between R3,850 and R8,899 per month
- ◯ Between R8,900 and R19,999 per month
- ◯ Between R20,000 and R29,999 per month
- ◯ Between R30,000 and R39,999 per month
- ◯ More than R40,000 per month
- ◯ Don't know / prefer not to say

In what province do you currently live?

- ◯ Eastern Cape
- ◯ Free State
- ◯ Gauteng
- ◯ Limpopo
- ◯ KwaZulu-Natal
- ◯ Mpumalanga
- ◯ Northern Cape
- ◯ North West
- ◯ Western Cape

In general, how would you rate your OVERALL health?

- ◯ Excellent
- ◯ Very good
- ◯ Good
- ◯ Fair
- ◯ Poor

What race/ethnicity best describes you?

- ◯ Asian/Indian
- ◯ Black African
- ◯ Coloured
- ◯ White
- ◯ Other
- ◯ Prefer not to say

Which political party do you support? (Please feel free to skip this question if you are not comfortable giving a response)

____

Do you live with an elderly person?

- ◯ Yes
- ◯ No
